# A test of the symbol interdependency hypothesis with both concrete and abstract stimuli

**DOI:** 10.1371/journal.pone.0192719

**Published:** 2018-03-28

**Authors:** Simritpal Kaur Malhi, Lori Buchanan

**Affiliations:** Department of Psychology, University of Windsor, Windsor, Ontario, Canada; Universita degli Studi di Roma La Sapienza, ITALY

## Abstract

In Experiment 1, the *symbol interdependency hypothesis* was tested with both concrete and abstract stimuli. Symbolic (i.e., semantic neighbourhood distance) and embodied (i.e., iconicity) factors were manipulated in two tasks—one that tapped symbolic relations (i.e., semantic relatedness judgment) and another that tapped embodied relations (i.e., iconicity judgment). Results supported the *symbol interdependency hypothesis* in that the symbolic factor was recruited for the semantic relatedness task and the embodied factor was recruited for the iconicity task. Across tasks, and especially in the iconicity task, abstract stimuli resulted in shorter RTs. This finding was in contrast to the concreteness effect where concrete words result in shorter RTs. Experiment 2 followed up on this finding by replicating the iconicity task from Experiment 1 in an ERP paradigm. Behavioural results continued to show a reverse concreteness effect with shorter RTs for abstract stimuli. However, ERP results paralleled the N400 and anterior N700 concreteness effects found in the literature, with more negative amplitudes for concrete stimuli.

## Introduction

Understanding the mechanism through which humans obtain meaning from words has been an ongoing pursuit for researchers in the area of psycholinguistics. Over the years, various theories have been proposed to explain how we understand words in general and how we understand concrete versus abstract words. A review of these theories and their associated empirical findings follows (see Table 1 for a summary). This will set the stage for the present study which will test the *symbol interdependency hypothesis* using a stimulus set that taps into concrete and abstract relationships in a novel way.

**Table 1:** Summary of word processing theories, with their basic tenets, predictions, and empirical evidence.

Theory	Basic Tenets	Predictions	Empirical Evidence
**Symbolic; Linguistic; Distributional Theories**	Word meaning is derived from the linguistic context in which the word occurs, i.e., how a target word relates to other words Recruitment of perceptual system is unnecessary	Lexical co-occurrence models (e.g., HAL, LSA, BEAGLE, LDA, Topic Model, HiDEx, and WINDSORS) correlate with human performance on psycholinguistic tasks	Buchanan et al., 2001; Burgess & Conley, 1998; Burgess & Lund, 1997; Foltz et al., 1998; Kintsch, 2000; Landauer & Dumais, 1997; Louwerse et al., 2006; Lund & Burgess, 1996; Siakaluk et al., 2003
**Embodied Cognition Theories**	Perceptual system is recruited when understanding words Mental simulations and imagery facilitate understanding	Shorter RTs for high body-object interaction words Shorter RTs when congruency between word/sentence meaning and motor movement required for response Processing advantage for words presented in their typical spatial locations Sensorimotor system is activated during word processing Impairments in verb processing for patients with motor neuron disease	Aravena et al., 2010; Aziz-Zadeh et al., 2006; Bak et al., 2001; Boulenger et al., 2008; Boulenger et al., 2009; Chasteen et al., 2010; Esopenko et al., 2012; Estes et al., 2008; Glenberg et al., 2008; Glenberg & Kaschak, 2002; Glenberg & Robertson, 1999; Guan et al., 2013; Hauk et al, 2004; Lugli et al., 2013; Meier & Robinson, 2004; Santana & de Vega, 2011; Setic & Domijan, 2007; Siakaluk et al., 2008; Schubert, 2005; Tettamanti et al., 2005; Wilson & Gibbs, 2007; Zhang et al., 2014; Zwaan & Taylor, 2006; Zwaan & Yaxley, 2003
**Integrated Theories: Symbol Interdependency Hypothesis (Louwerse, 2007); Representational Pluralism (Dove, 2009); Language and Situated Simulation Theory (Barsalou et al., 2008)**	Word meaning is derived by accessing both symbolic and embodied information The relative influence of either symbolic or embodied information depends on task requirements Symbolic factors are more important earlier on in word processing Language encodes perceptual information	Tasks with a linguistic focus, e.g., semantic relatedness judgments, will highlight the role of symbolic information and tasks with an embodied focus, e.g., iconicity judgments, will highlight the role of embodied information Symbolic factors will be more important for shallow tasks and embodied factors will play a role in tasks involving deeper processing	Louwerse & Connell, 2011; Louwerse & Hutchinson, 2012; Louwerse & Jeuniaux, 2010; Simmons et al., 2008
**Dual Coding Theory (Paivio, 1971)**	Concrete words have a processing advantage because they activate both the linguistic (verbal) and imagistic (nonverbal) systems, whereas abstract words only activate the linguistic (verbal) system	Shorter RTs for concrete words compared to abstract words	Binder et al., 2005; Ernest & Paivio, 1971; Levine & Banich, 1982; Shibaraha & Lucero-Wagoner, 2002; Wang et al., 2010
**Context Availability Theory (Schwanenflugel et al., 1988; Schwanenflugel & Shoben, 1983; Schwanenflugel & Stowe, 1989)**	Concrete words have more easily accessible and richer contextual information	Shorter RTs for concrete words compared to abstract words	Laszlo & Federmeier, 2011

### Symbolic theory

Language comprehension has been explained through symbolic—also referred to as linguistic, distributional, computational, or amodal—theories [1]. We are not considering symbolic approaches to cognition in general, but rather, we are using a constrained definition of symbolic theory here to discuss a particular type of symbolic theory relevant to the semantic processing literature. Symbolic theories of language maintain that words, considered to be an external medium, map onto internal symbolic representations of word meaning [2]. There is an arbitrary relationship between symbols and what they represent in the real world, and the meaning of a linguistic symbol is understood by how it is related to other linguistic symbols [3]. Thus, words are understood via rule-governed manipulation of symbols [4]. Notably, perceptual inputs are transduced into symbols so that the process of understanding words does not necessitate perceptual experience nor does it recruit the brain’s sensorimotor system [3–4]. In other words, sophisticated capacities such as language comprehension are viewed as being different from lower level perceptual processes [5]. It is important to note that proponents of the symbolic theory do not necessarily agree on the features described, i.e., whether or not symbols are used, whether word meanings are inferred from experience, and whether people rely on rules. There is considerable variation in how the symbolic theory is defined, and as such, we hope to provide a description that captures the essence of the symbolic theory as opposed to providing a unitary definition of the theory.

Collins and Quillian [6] introduced a symbolic, hierarchical model of semantic knowledge in which concepts were represented as nodes, with general concepts (e.g., *animal*) located at the top of the hierarchy, and more specific concepts (e.g., *robin*) located at the bottom. Collins and Loftus [7] revised the earlier hierarchical model by introducing a spreading activation model wherein concept activation proceeds or spreads from the target concept to related concepts. Both the hierarchical and the spreading activation model assume localist representation such that each concept corresponds to a single node. On the other hand, in distributed representation models [8], concepts are represented as unique patterns of activation among common nodes. Distributed representation models also symbolize concepts through the activation of representations of the individual features of the concept, e.g., connectionist feature-based approaches to semantic memory [9].

While some symbolic views of language are feature-based approaches, other symbolic views of language are use-based approaches that rely on statistical regularities. As such, researchers from the symbolic orientation have aimed to capture the meaning of words by computationally studying word usage in large bodies of text. Computational analyses have been used to develop lexical co-occurrence models. One such co-occurrence model is Hyperspace Analogue to Language (HAL) [10]. In HAL, the different contexts in which a word appears in a large body of text are analyzed and meaning is derived from the number of times that certain pairs of words co-occur. Words are represented in the form of vectors in a high-dimensional semantic space. In this semantic space, word vectors with smaller distances between them are considered to be more similar in meaning than word vectors located farther apart. Consistent with the symbolic view, the meaning of a word is obtained from its relationship to other words as opposed to the referent of the word. For example, the word *flower* is understood because it is related to other words such as *plant*, *garden*, and *nature*. These latter words are considered to be the semantic neighbours of *flower*. Other lexical co-occurrence models include Latent Semantic Analysis (LSA) [11], Bound Encoding of the Aggregate Language Environment (BEAGLE) [12], Latent Dirichlet Allocation (LDA) [13], Topic Model [14], and High Dimensional Explorer (HiDEx) [15]. Although there are subtle differences among these models, the overarching commonality is that word meaning is derived through an analysis of the words that a target word associates with at either the sentence level or in some larger context. Unfortunately, co-occurrence in both HAL and LSA is influenced by word frequency such that two words with a high frequency are more likely to co-occur by chance than are two words with a low frequency. This is unfortunate because it makes the metrics derived from these models less useful in psycholinguistic experiments because frequency is a confound. As psycholinguistic tasks are highly sensitive to frequency effects, spurious frequency effects may hide less robust co-occurrence effects. Durda and Buchanan [16], however, removed the influence of word frequency by obtaining frequency-free measures of word co-occurrence (using log-relative frequency ratios to address high-frequency values and scaling procedures to address low-frequency values; a description of these technique is beyond the scope of this paper and those interested are referred to Durda and Buchanan [16] for the algorithm) and introduced an adaptation of HAL called WINDSORS (Windsor Improved Norms of Distance and Similarity of Representations of Semantics).

Lexical co-occurrence models produce results that correlate with human performance on various psycholinguistic tasks [2,10,11,17–22]. To summarize, symbolic views of word meaning based on lexical co-occurrence models understand meaning as being derived from the linguistic context in which the word occurs. A number of models have been introduced over the years and they differ with respect to how the linguistic units are assumed to be represented but in all cases the representations are, in some way, a reflection of the linguistic context.

### Embodied cognition theory

Symbolic theories can be contrasted with embodied theories, also referred to as perceptual or modal theories. Historically, this etymological debate between conventionalism (i.e., symbolic) and naturalism (i.e., embodied) traces back to Plato’s [23] *Cratylus*. In conventionalism, names are arbitrarily adopted with local or national convention determining which names are attached to objects. In naturalism, names are adopted in a specific way, such that names encode descriptions of their objects. Embodied theories maintain that language comprehension is grounded in sensorimotor interactions with the environment. It is important to note that embodied theories range on a continuum of being weakly embodied to strongly embodied (the interested reader is referred to Meteyard et al. [3] for a discussion of this continuum). In contrast to the symbolic view, real world perceptual experiences as opposed to symbolic representation form the basis of understanding words. Returning to the *flower* example, the embodied theory would suggest that we understand this word through our experience of seeing, touching, and smelling flowers, whereas from a symbolic co-occurrence perspective, one need not have actual experience with a flower to understand its meaning. This is not to say that symbolic theories are nativist. With symbolic theories, linguistic experience, rather than perceptual experience forms the basis of understanding words. Barsalou [24], in his *perceptual symbols systems theory*, states that during direct perceptual experience, sensorimotor regions of the brain are activated in a bottom-up fashion. Perceptual symbols, or representations of the experience, then become encoded in the brain. Later, sensorimotor regions of the brain are partially reactivated in a top-down manner in the absence of direct perceptual experience. That is, when words are encountered, a mental simulation occurs and that indirect experience facilitates comprehension. Similarly, Glenberg and Robertson [25] proposed the *indexical hypothesis*, which states that sentences are understood by simulating the actions that underlie them. Thus, the embodied cognition theory proposes that mental imagery activates sensorimotor systems [26–27] and that imagery and action have shared neural substrates [28].

Numerous studies have provided support for the embodied view of language [25,29–35]. At the level of individual words, researchers have found a *body-object interaction (BOI) effect* [33]. Words with a high BOI, that is, words whose referents with which the body can physically interact with ease, facilitate responding on lexical and phonological decision tasks when compared to words with a low BOI. At the level of sentences, Glenberg and Kaschak [30] found an interaction between performing an action and sentence comprehension which they coined the *action-sentence compatibility effect* (ACE). The embodied view of language has also gained support from event-related potential (ERP) [36–37], neuroimaging [38–42], and patient [43–44] studies.

#### Iconicity

Iconicity has been used to support the embodied cognition theory. Iconicity occurs when a linguistic symbol matches its referent. There are different forms of iconicity, e.g., onomatopoeia represents an auditory form of iconicity when words sound like their referent. Spatial iconicity, hereafter referred to as iconicity, has been the focus of prior research and refers to when the spatial positions of words match how their referents appear. In research, this is whether the relative positions of words on a computer screen match the relative positions of their referents [45]. Studies of iconicity find a processing advantage for words that are spatially presented in a manner that reflects their meaning. According to the embodied theory, there is a processing advantage for words presented in their referents’ typical locations because of our sensorimotor history with such an arrangement in our world. For example, Setic and Domijan [46] found that RTs for judging the names of flying animals were shorter when displayed at the top of a computer screen and names of non-flying animals were judged faster when displayed at the bottom of a computer screen. These results were replicated when the names of animals were replaced with non-living things typically associated with either upper or lower space. Similarly, Estes, Verges, and Barsalou [47] found that words representing objects associated with high or low space stalled subsequent identification of unrelated visual targets presented in the object’s typical location. This ability for the meaning of a word to orient spatial attention has been referred to as conceptual cuing [48]. Zwaan and Yaxley [45] demonstrated the iconicity effect with word pairs. Participants saw word pairs either in an iconic relationship (e.g., the word *attic* presented above the word *basement*) or in a reverse-iconic relationship (e.g., the word *basement* presented above the word *attic*) and were asked to indicate whether the two words were semantically related. Results revealed that RTs were shorter when word pairs were displayed in an iconic relationship compared to when word pairs were displayed in a reverse-iconic relationship. This iconicity effect disappeared when the word pairs were presented horizontally.

While concrete word pairs have been investigated in the context of iconicity, i.e., Zwaan and Yaxley [45], abstract word pairs have yet to be studied. However, at the level of individual words, iconicity has been demonstrated with both concrete and abstract stimuli. For example, when participants are asked to judge which of two social groups (e.g., *masters* and *servants*) have more power, RTs are shorter when the more powerful group is displayed at the top of the screen. Conversely, when asked to judge which group has less power, RTs are shorter when the less powerful group is at the bottom of the screen [49]. Moreover, when participants are asked to make evaluations of words presented on a computer screen, evaluations of positive words are faster when the words are displayed at the top of the screen, whereas evaluations of negative words are faster when the words are displayed at the bottom of the screen [50]. Positive evaluations also tend to activate higher areas of visual space and negative evaluations activate lower areas of visual space [50]. Zhang, Hu, Zhang, and Wang [51] primed participants with either up or down arrows and then presented them with neutral words or target emotional words that were either positive (e.g., *happy*) or negative (e.g., *sad*). Results showed that N400 amplitudes were greater when target words were primed by incongruent spatial information (e.g., up arrow priming the word *sad*). In addition to the top and the bottom of the screen, the right and the left of the screen also activate positive and negative associations, respectively [52]. These findings can be explained by the *conceptual metaphor theory* in which concepts are embedded in spatial relations (e.g., *up* represents power and happiness) [53–54].

### Integrated theories

While symbolic and embodied theories tend to be viewed as being at odds with one another, historical and recent attempts to integrate these theories have been documented [55]. Paivio’s [56] *dual coding theory* advocated for separate cognitive subsystems for verbal and nonverbal information. According to the *dual coding theory*, depending on task requirements, one or multiple types of processing would be activated. Another proposal attempting to integrate symbolic and embodied theories is Dove’s [57] *representational pluralism*, in which the meaning of a word results from diverse semantic codes. Some codes are perceptual (i.e., embodied, modal) and others are non-perceptual (i.e., symbolic, amodal). Therefore, for any given word, both sensorimotor simulations and linguistic representations are activated [58]. Similarly, the *language and situated simulation theory* [59] proposes that language (symbolic factors, e.g., co-occurrence) and situated simulation (embodied factors, e.g., iconicity) both play a role in conceptual processing. This theory incorporates a temporal component such that both symbolic and embodied factors are activated immediately, but symbolic activation reaches its peak earlier than embodied activation [60–62].

#### Symbol interdependency hypothesis

Louwerse [63] proposed the *symbol interdependency hypothesis*, in which the linguistic system serves as a shortcut to the perceptual system. According to Louwerse [64],

Language has evolved to become a communicative short-cut for language users and encodes relations in the world, including embodied relations. The symbol interdependency hypothesis thus emphasizes the importance of the language structures, without discarding the notion of symbol grounding…. To facilitate this process, language is organized in such a way that language encodes perceptual information.

(p. 7)

Language comprehension for the most part uses symbolic representation and the embodied representations of words do not necessarily need to be accessed or fully activated. Moreover, when symbolic processing is sufficient for the task at hand, embodied factors (e.g., iconicity) may not be recruited. As the linguistic system evolved later than the simulation system, it does not necessarily provide access to deep conceptual information. While embodied information enables a thorough understanding of words, symbolic information is more efficient and is adequate for providing most meaning. Symbolic factors (e.g., co-occurrence) are believed to be less precise than embodied factors, providing quick approximate representations, which the perceptual system then refines. Parallel to the *language and situated simulation theory*, symbolic factors are proposed to be more important earlier on in word processing. This has been linked to depth of processing with symbolic factors most important for shallow tasks, and embodied factors coming into play for tasks involving deeper processing.

Louwerse and Jeuniaux [65] found support for the *symbol interdependency hypothesis* in a study in which participants made speeded judgments about semantic relatedness or iconicity for word pairs or pictures. The symbolic factor was operationalized as frequency of word order, that is, whether word pairs were presented in the order in which they typically occur in language, and the embodied factor was operationalized as iconicity, that is, whether word pairs were presented in the spatial relationships in which their referents typically occur. An analysis of RTs and error rates revealed that the symbolic factor dominated in the semantic relatedness task for word pairs (the more shallow processing) and the embodied factor dominated in the iconicity task for pictures (the deeper processing).

In summary, integrated theories argue that meaning is derived from words by accessing both symbolic and embodied information. However, the relative influence of either symbolic or embodied information depends on task requirements. The *symbol interdependency hypothesis* proposes that tasks with a linguistic focus, e.g., semantic relatedness judgments, highlight the role of symbolic information and tasks with an embodied focus, e.g., iconicity judgments, highlight the role of embodied information.

### Theories of concrete and abstract word processing

Concrete words (e.g., *apple*) are words that have direct sensory referents and words that can be easily visualized. Abstract words (e.g., *respect*), in contrast, are words without such characteristics [66]. Concreteness is related to a variable known as imageability, which is defined as how easily a word can conjure a visual image. While concreteness and imageability are related, they are not identical, as the definition of concreteness also includes whether the referent of a word can be situated in time and space [66]. Many studies have found a concreteness effect [67], whereby when presented with both concrete and abstract stimuli, participants more quickly recognize [68] and better remember [56] concrete stimuli compared to abstract stimuli. Concrete words have also been found to be better preserved after neurological impairment [69–72]. The concreteness effect has been explained by various theories. The *dual coding theory* [56] explains the concreteness effect in terms of the type of information available. That is, concrete words have a processing advantage because they activate both the linguistic (verbal) and imagistic (nonverbal) systems, whereas abstract words only activate the linguistic (verbal) system. For example, participants produce comparable RTs for concrete and abstract words when asked to generate word associates. However, they produce shorter RTs for concrete words than abstract words when asked to generate mental imagery [73]. The *dual coding theory* has received empirical support from visual field studies which demonstrate that concrete words presented to the left visual field (right hemisphere, which is dominant for visual processing) are processed faster than those presented to the right visual field [74–75]. Imaging studies also provide support for the *dual coding theory* as areas involved in perception and imagery have more activation for concrete compared to abstract words [76–77]. On the other hand, the *context availability theory* [66,78–79] explains the concreteness effect with respect to how much information is available. According to this theory, concrete words are strongly associated with a few contexts, whereas abstract words are weakly associated with many contexts. Concrete words thus have more easily accessible and richer contextual information, which facilitates processing [80]. Another theory to explain the concreteness effect and one that integrates the *dual coding theory* with the *context availability theory* is the *context extended dual coding theory* [81]. This theory proposes that concrete words have a processing advantage because of both their ability to generate mental images as well as more semantic activity within a verbal system.

A problem with studies that examine concrete and abstract word processing is that when studying concrete and abstract words at the individual level, both types of words can be visualized. In the case of concrete words, one visualizes the word itself, and in the case of abstract words, one visualizes the word indirectly by visualizing a referent, e.g., visualizing a church for the word *religion*. Thus, a fundamental problem in research on concrete and abstract word processing is the concretizing of abstract words. Directly visualizing concrete words has been argued to facilitate processing by the *dual coding theory* [56] and being unable to visualize abstract words has been argued to slow down its processing. While this is reasonable, the confound is that participants may be indirectly visualizing abstract words and it is this indirect visualization that is slowing down processing, rather than not visualizing the abstract words at all. Thus, what appears to be a concreteness effect is confounded by the concretizing of abstract words.

### The present study

The first major objective of the present study was to test the *symbol interdependency hypothesis* with both concrete and abstract stimuli. To our knowledge, abstract word pairs have not been incorporated into an iconicity task before. We also developed a stimulus set that taps into abstract relationships in a novel way. Specifically, we attempted to investigate the difference between concrete and abstract stimuli while preventing the concretizing of abstract words. In developing our stimulus set, it was ensured that, according to their definitions, concrete words (e.g., *nose*–*tongue*) were easily visualized, while abstract words (e.g., *accept—reject*) were not [66]. This was possible by activating the relationship between the word pairs as opposed to activation at the level of the individual words. Participants were required to attend to the abstract relationship between the individual words rather than attending to the abstract words themselves. Therefore, the present study attempted to test the symbol interdependency hypothesis with both concrete and abstract word pairs and developed a pure measure of abstractness to help circumvent the confound of concretizing abstract words.

The second major objective of the present study was to extend supportive findings from the *symbol interdependency hypothesis* to a novel symbolic factor. Previous research [65] used the order in which words typically occur in language as a symbolic factor and iconicity as an embodied factor. Consistent with previous research [65], the present study used the same embodied factor, i.e., iconicity. While the format of iconic or reverse-iconic information is not in and of itself sensory or embodied, seeing words presented in such a format activates their corresponding perceptual representations and thus has been used as a proxy of embodiment. The present study used a novel symbolic factor, i.e., semantic neighbourhood distance between word pairs. Distance between semantic neighbours was determined by the WINDSORS lexical co-occurrence model, an adaptation of HAL controlling for word frequency [16]. The present study used the same tasks, i.e., semantic relatedness and iconicity judgments, as Louwerse and Jeuniaux [65]. Based on the research described above, we predict that the symbolic factor (i.e., word pairs that are close semantic neighbours versus distant semantic neighbours) would be more important for the semantic relatedness task, and the embodied factor (i.e., word pairs that are in an iconic relationship versus a reverse-iconic relationship) would be more important for the iconicity task. Consistent with the literature on concrete and abstract word processing, we predict that, across tasks, and especially for the iconicity task (more imagery-based task), concrete word pairs (e.g., *desk—carpet*) will result in shorter RTs compared to abstract word pairs (e.g., *beauty—ugly*).

## Experiment 1

### Method

#### Participants

The study was approved by the University of Windsor Research Ethics Board (REB #: 31436). Participants were recruited from July 17, 2014 to September 22, 2016. Eighty (n = 40 for the semantic relatedness task and n = 40 for the iconicity task; 19 males, 61 females, *M*_*age*_ = 20.7 years, age range: 18–42 years) University of Windsor undergraduate students participated for partial course credit. All participants were at least 18 years of age, had learned English as their first language, and had normal or corrected-to-normal vision.

#### Materials

The stimulus set is presented in S1 Appendix. The stimulus set was developed using WINDSORS [16] and Wordmine2 [82]. The symbolic factor was operationalized through semantic neighbours, with close semantic neighbours defined as less than 50 words away from one another, and distant semantic neighbours defined as greater than 200. Semantic neighbourhood distance was an ordinal measurement with the target word located X words away from its neighbour of interest. For example, *nose* is the 9^th^ neighbour of *tongue* and *tongue* is the 22^nd^ neighbour of *nose*. Consistent with Louwerse and Jeuniaux [65], the embodied factor was operationalized through iconicity, i.e., whether word pairs were presented in the spatial relationships in which their referents typically occur or whether these relationships were reversed. Consistent with Schwanenflugel and Stowe [66], concreteness was operationalized as stimuli with direct sensory referents and that could be easily visualized, while abstractness was operationalized as stimuli without direct sensory referents and that could not be easily visualized.

Orthographic frequency values were restricted to a range of 10–200. An ANOVA was conducted to ensure that the target word pairs’ average orthographic frequencies [*F*(1, 79) = 1.33, *p* = .25] and average number of letters [*F*(1, 79) = 2.06, *p* = .059] did not differ across conditions. To avoid an alliteration effect, no two words in the pairs began with the same letter. An ANOVA was also conducted to ensure that semantic neighbourhood distance did not differ between the concrete and abstract stimuli [*F*(1, 79) = .35, *p* = .55]. Age of acquisition was the older age associated with the word pair. For example, for the word pair *flower-vase*, the word *flower* is acquired at age 3.11 and the word *vase* is acquired at age 7.89, thus the age of acquisition for the entire word pair was entered as 7.89. As expected, the age of acquisition [83] for concrete words pairs differed from the age of acquisition for abstract word pairs [*F*(1, 79) = 14.048, *p <* .001], such that abstract word pairs were acquired at a later age. The means and standard deviations (SDs) for orthographic frequencies, number of letters, and age of acquisition per condition are displayed in Table 2. Half of the target word pairs were close semantic neighbours and half were distant semantic neighbours. Moreover, half of the close and distant semantic neighbours were presented in an iconic relationship and half were presented in a reverse-iconic relationship. The stimulus set was counterbalanced so that the word pairs were presented in both iconic and reverse-iconic form, with no participant seeing the same word pair in both iconic and reverse-iconic form. The stimulus set contained 40 concrete word pairs and 40 abstract word pairs. The stimulus set for the semantic relatedness task also contained 80 filler word pairs with no semantic relationship, as measured by WINDSORS [16]. Like the target word pairs, no two filler word pairs began with the same letter. Number of letters did not differ between the filler words and the target words [*F*(1, 317) = 1.24, *p* = .27]. Filler word pairs included both concrete and abstract words. The filler word pairs are presented in S2 Appendix.

**Table 2:** Means and SDs for frequency, word length, and age of acquisition (AoA) per condition in the stimulus set.

Condition	Frequency	Word Length	AoA
**Abstract-Close**	44.81(17.65)	12.15(2.68)	7.68(2.07)
**Abstract-Distant**	41.73(20.07)	11.9(3.23)	8.12(1.67)
**Concrete-Close**	37.81(28.14)	10.9(2.17)	6.15(1.55)
**Concrete-Distant**	35.14(23.09)	10(1.86)	6.19(1.39)

#### Procedure

The procedure was described to participants and written informed consent was obtained. The experiment was run on a PC in an individual testing room using DirectRT [84]. Word pairs were presented in a vertical position in the middle of a black background, with a distance of less than one inch between the middle of the two words. Stimuli were presented in all capital letters, with size 24 turquoise coloured Times New Roman bold-faced font. Each word pair appeared one at a time in random order and remained on the screen until the participant gave their response by pressing either the “z” key or the “/” key which were covered by “yes” and “no” stickers to simplify responding. These response keys were counterbalanced across participants to avoid any confound of dominant hand responding. Participants were randomly assigned to either the semantic relatedness task or the iconicity task. Participants first completed a practice session with four trials, which had two concrete items and two abstract items not on the experimental list. The practice session included corrective feedback. For the semantic relatedness task, participants were asked to indicate whether the pair of words was related in meaning or not by pressing the “yes” key if the word pair was related (e.g., *stove—oven*) and pressing the “no” key if the word pair was unrelated (e.g., *book—snow*). Participants were advised that their judgments should be intuitive, that they should not have to think of ways to relate the words, and that when word pairs were unrelated, they would not bear any obvious relationship to one another. For the iconicity task, participants were asked to indicate whether the positions of the words matched how their referents appear, either in everyday objects (for concrete words), or in relationships (for abstract words) by pressing the “yes” key if the word pair was iconic (e.g., *stove—oven*) and pressing the “no” key if the word pair was reverse-iconic (e.g., *oven—stove*). For concrete words, participants were given the example of *pot* and *plant*, where one would expect to see a *plant* above a *pot*. For abstract words, participants were given the example of *doctor* and *patient*, where because of their greater authority and power, *doctor* would be above *patient*. To illustrate the different kinds of abstract relationships, participants were also given the example of *happy* and *sad*, where because of its positive and uplifting associations, *happy* would be above *sad*. Participants were advised not to make moral judgments and instead, to consider how concepts stereotypically appear. For both the semantic relatedness task and the iconicity task, participants were informed that reaction times were being measured and that they should use both index fingers to make their responses as quickly as possible, however, not at the expense of accuracy. Task instructions are presented in S3 Appendix.

### Results

Only responses to target word pairs were included in the analysis. A minimum accuracy rate of 70% was used for both participants and words. This resulted in the removal of responses for two concrete word pairs and four participants. The analyses were performed on the remaining data. All incorrect responses, as well as responses faster than 300 ms, were removed, resulting in the removal of 619 observations (11.7% of the remaining data).

Data was analyzed using R [85] and the lmerTest package [86]. Correct responses were analyzed in a linear mixed effects analysis. RTs were log transformed. With respect to collinearity, variance inflation factors for all coefficients were equal to 1. As fixed effects, the factors task, response key, concreteness, iconicity, and semantic neighbours were entered into the model. As random effects, subjects and items were entered into the model. The model was fitted with random slopes for task by item and random slopes for concreteness and iconicity by subject. After the model was fitted, data was trimmed using the LMERConvenienceFunctions package [87]. Outliers with a standardized residual at a distance greater than 2.5 standard deviations from 0 were excluded. This resulted in the removal of 139 observations (2.6% of the data). Participant mean logged RTs, SDs, and error rates per condition for the final data set are displayed in Table 3 for the semantic relatedness task and Table 4 for the iconicity task.

**Table 3:** Mean log RTs (with SDs) and average error rates per condition for the semantic relatedness task.

Condition	Mean Log RT (ms)	Average Error Rate (%)
**Abstract-Close-Iconic**	6.9 (.31)	3.33
**Abstract-Close-Reverse Iconic**	6.9 (.33)	3.33
**Abstract-Distant-Iconic**	7.05 (.32)	9.74
**Abstract-Distant-Reverse Iconic**	7.04 (.31)	11.54
**Concrete-Close-Iconic**	7 (.34)	7.44
**Concrete- Close-Reverse Iconic**	6.99 (.33)	7.44
**Concrete-Distant-Iconic**	7.14 (.35)	17.38
**Concrete-Distant-Reverse Iconic**	7.13 (.37)	18.23

**Table 4:** Mean log RTs (with SDs) and average error rates per condition for the iconicity task.

Condition	Mean Log RT (ms)	Average Error Rate (%)
**Abstract-Close-Iconic**	7.24 (.46)	7.59
**Abstract-Close-Reverse Iconic**	7.4 (.45)	10.54
**Abstract-Distant-Iconic**	7.3 (.4)	5.15
**Abstract-Distant-Reverse Iconic**	7.46 (.4)	11.35
**Concrete-Close-Iconic**	7.62 (.44)	8.38
**Concrete- Close-Reverse Iconic**	7.77 (.42)	16.8
**Concrete-Distant-Iconic**	7.63 (.45)	11.11
**Concrete-Distant-Reverse Iconic**	7.77 (.43)	19.52

**Fig 1:**
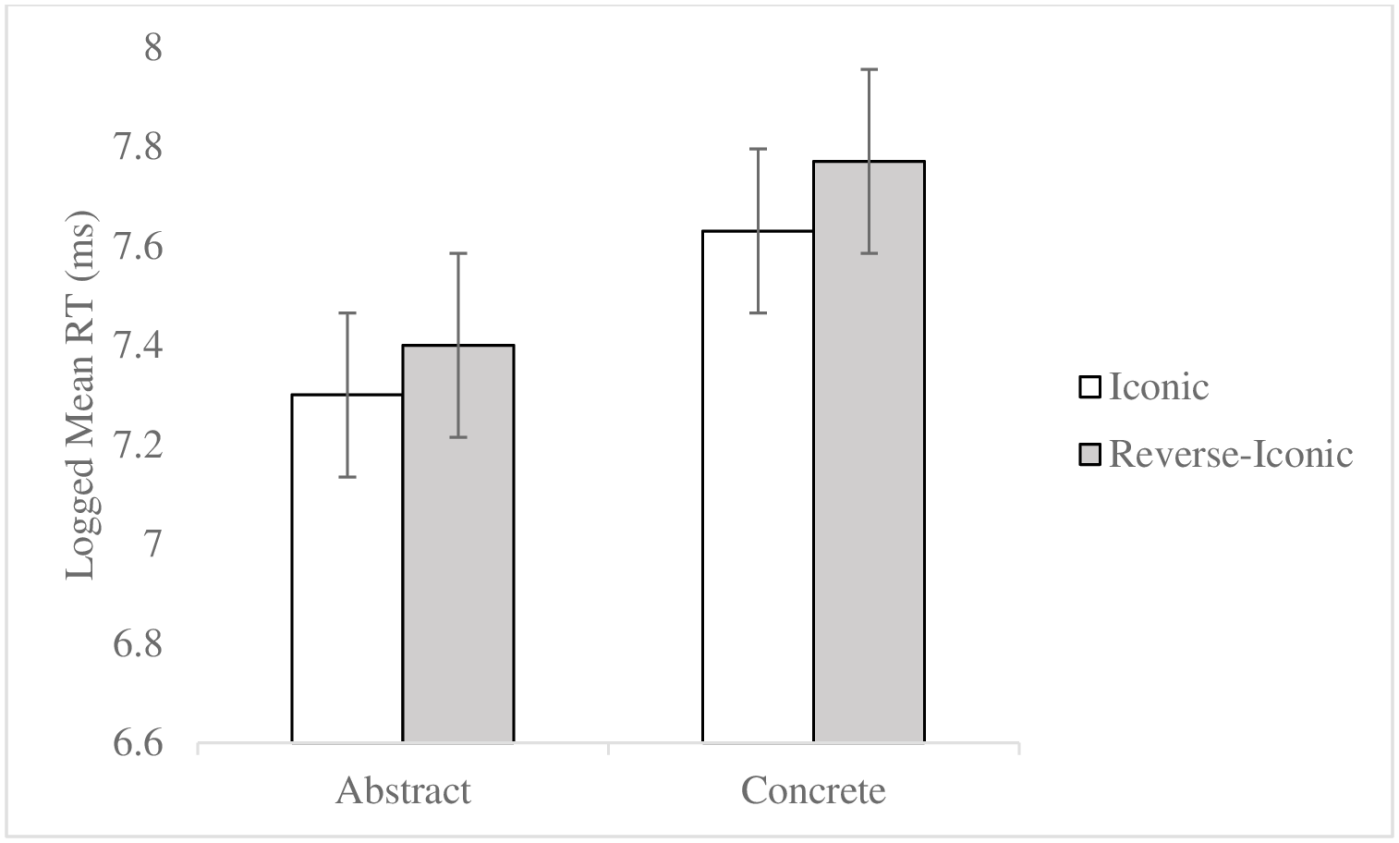
Embodied factor in the iconicity task. Error bars represent standard error.

**Fig 2:**
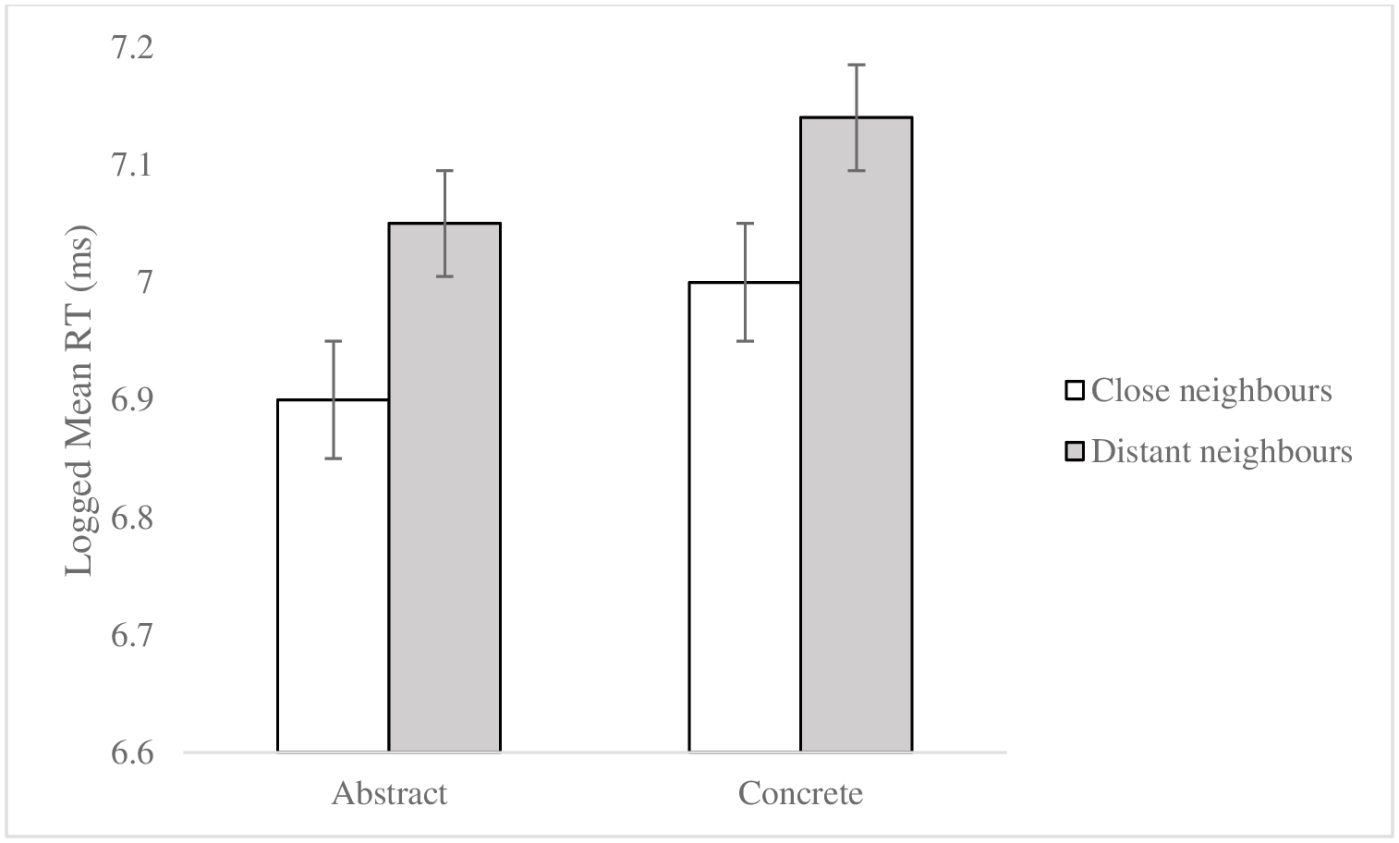
Symbolic factor in the semantic relatedness task. Error bars represent standard error.

P-values were obtained for the fixed effects using the lmerTest package with Satterthwaite approximations to degrees of freedom [86]. There was no main effect of response key [*t*(73) = -.9, *p* = .37]. There was an interaction between task and concreteness [*t*(4746) = 12.75, *p <* .001], task and iconicity [*t*(4734) = 5.48, *p <* .001], and task and semantic neighbours [*t*(4744) = -2.55, *p* = .011]. Post-hoc analyses with Tukey’s adjustment for multiple comparisons were performed using the lsmeans package [88]. For the interaction between task and concreteness, post-hoc analyses showed that in the iconicity task, the effect of abstract word pairs resulting in shorter RTs compared to concrete word pairs [*t*(83) = 8.35, *p <* .001] was larger than that observed in the semantic relatedness task [*t*(87) = 2.9, *p* = .0047]. For the interaction between task and iconicity, post-hoc analyses showed that in the iconicity task, iconic word pairs resulted in shorter RTs compared to reverse-iconic word pairs [*t*(4633) = 12.63, *p <* .001] (Fig 1), however in the semantic relatedness task, iconic word pairs did not result in shorter RTs compared to reverse-iconic word pairs [*t*(4585) = -.66, *p* = .51]. For the interaction between task and semantic neighbours, post-hoc analyses showed that in the semantic relatedness task, close semantic neighbours resulted in shorter RTs compared to distant semantic neighbours [*t*(75) = 4.58, *p <* .001] (Fig 2), however in the iconicity task, close semantic neighbours did not result in shorter RTs compared to distant semantic neighbours [*t*(77) = .39, *p* = .0037].

For accuracy, random subject and item effects were analyzed using a mixed logit model (generalized linear mixed model) for the binomial dependent variable (i.e., correct or incorrect) [89]. The model was fitted with random slopes for task and iconicity by item and random slopes for concreteness and iconicity by subject. The optimizer bobyqa was used to fit the model. There was no main effect of response key [*z* = .074, *p* = .94]. There was a main effect of concreteness [*z* = -2.57, *p* = .01], with concrete stimuli increasing the likelihood of making an error by 1.69 times ± .23 (standard errors) compared to abstract stimuli. There was an interaction between task and iconicity [*z* = 4, *p <* .001] and an interaction between task and semantic neighbours [*z* = -3.28, *p* = .001]. For the interaction between task and iconicity, post-hoc analyses showed that in the iconicity task, reverse-iconic word pairs resulted in more errors compared to iconic word pairs [*z* = 5.21, *p <* .001], however in the semantic relatedness task, reverse-iconic word pairs did not result in more errors compared to iconic word pairs [*z* = .19, *p* = .85]. For the interaction between task and semantic neighbours, post-hoc analyses showed that in the semantic relatedness task, distant semantic neighbours resulted in more errors compared to close semantic neighbours [*z* = 3.9, *p <* .001], however in the iconicity task, distant semantic neighbours did not result in more errors compared to close semantic neighbours [*z* = .96, *p* = .34].

### Discussion

The primary purpose of this study was to test the *symbol interdependency hypothesis* and to integrate symbolic and embodied approaches to language processing. The present study extends the existing literature by considering both concrete and abstract stimuli and by operationalizing the symbolic factor used to test the *symbol interdependency hypothesis* in an alternative way (i.e., semantic neighbours from WINDSORS). As predicted, results from both RTs and errors mapped onto the *symbol interdependency hypothesis*. The symbolic factor (i.e., semantic neighbourhood distance) was recruited for the task tapping symbolic relations (i.e., semantic relatedness task) and the embodied factor (i.e., iconicity) was recruited for the task tapping embodied relations (i.e., iconicity task). Therefore, this study adds to the large number of studies that conclude that language comprehension is fundamentally embodied by arguing that task can modulate the extent to which symbolic and embodied factors explain language comprehension.

In contrast to our predictions, an interesting pattern of results emerged with respect to the concrete and abstract stimuli. That is, across tasks, and especially for the iconicity task, abstract word pairs, e.g., *joy*–*sorrow*, contributed to shorter RTs compared to concrete word pairs, e.g., *shirt—pants*. This finding is unexpected in light of the well-known concreteness effect [67]. The reverse concreteness, or abstractness, effect is a novel finding with respect to word pairs. However, it has been reported at the level of individual words [90–92]. One possibility for the reverse concreteness, or abstractness, effect found in the present study is that the nature of these tasks may have provided an advantage for responding to the abstract stimuli. In the semantic relatedness task, it may have been easier for participants to judge the relatedness of opposite word pairs which were more prevalent within the abstract stimuli, e.g., *joy*–*sorrow* and *fast—slow*, as opposed to concrete word pairs that were related but not opposites, e.g., *shirt—pants* and *sky—grass*. In the iconicity task, it may have been easier for participants to judge the iconicity of abstract word pairs because these could not be visualized. While visualization may facilitate single word processing of concrete words in other tasks, in the present task, visualizing the concrete words slowed down RTs. The iconicity task can be conceptualized as involving two steps for concrete stimuli and one step for abstract stimuli. With concrete word pairs, the first step is visualization and the second step is mental manipulation. In contrast, with abstract word pairs, there is only the single step of mental manipulation. While abstract words can be concretized and consequently visualized, in our stimulus set, we were able to avoid this by activating the relationship between the word pairs as opposed to activating the individual words. Even at the level of the individual words, mean imageability ratings for the concrete word pairs were found to be higher than mean imageability ratings for the abstract word pairs [*F*(1, 32) = 87.05, *p <* .001] [93–96]. Concreteness and imageability have been reported to be highly correlated, with imageability accounting for 72% of the variability in concreteness [91]. As RTs were shorter for abstract stimuli in our study, this provides support for our proposal that the concretizing of abstract words through visualization of concrete associates contributes to longer RTs in research on concrete and abstract single word processing. If participants were not taking a visualization approach to the abstract word pairs in the iconicity task, what approach did they use? One possibility is that for the abstract word pairs, participants used an emotional valence approach, in which they tagged upper and lower space with emotions. Using the emotional valence approach, more emotionally valenced (more pleasant) words may have been tagged with upper space and less emotionally valenced (less pleasant) words may have been tagged with lower space. This was supported by greater differences in emotional valence among the abstract word pairs compared to the concrete word pairs [*F*(1, 73) = 66.28, *p <* .001] [97]. Overall, when judging iconicity, for the concrete word pairs, participants may have been taking a time-costly (two-steps) visualization approach, and for the abstract word pairs, participants may have been taking a time-efficient (one step) emotional valence approach. Such findings are consistent with Vigliocco, Meteyard, Andrews, and Kousta’s [98] *theory of embodied abstract semantics*, in which sensory-motor information contributes to understanding of concrete words and emotional information contributes to understanding of abstract words. Similarly, others [99–100] have suggested that whereas concrete concepts evoke more perceptual properties, abstract concepts evoke more properties that are situational and introspective.

Experiment 2 sought to investigate the neural underpinnings of the reverse concreteness, or abstractness, effect by replicating the iconicity task from Experiment 1 in an ERP paradigm. As the abstractness effect was greater in the iconicity task relative to the semantic relatedness task, we sought to focus our investigation only on the iconicity task. Moreover, our proposal that abstract words were processed faster because they did not evoke imagery was restricted to the iconicity task. Imagery was not part of our explanation for the abstractness effect in the semantic relatedness task. Many studies have investigated concreteness using ERPs (see [101] for a review). ERP studies report a greater N400 (300–500 ms) amplitude for concrete words compared to abstract words, with this finding most prominent at central and posterior electrode sites [81,102–108]. ERP studies also report a greater N700 (500–800 ms) amplitude for concrete words compared to abstract words, with this finding most prominent at anterior electrode sites [81,104–105,108–110]. Researchers have conceptualized the anterior N700 as an index of imagery [108,111–112].

West and Holcomb [108] used a sentence verification task where the final word of a sentence was either concrete or abstract. There were three conditions, with the verification involving generating an image, making a semantic decision, or evaluating the surface characteristics of the word (i.e., whether a probe letter was present in the target word). These researchers found N400 and anterior N700 concreteness effects only in the image generation and semantic decision conditions, with the anterior N700 effect most robust in the imagery task. This led them to conceptualize the anterior N700 as an index of imagery. In a related study, Gullick, Mitra and Coch [111] asked participants to either decide whether it was easy to make a mental image for a word or to make a decision about the word’s surface characteristics. These researchers found a larger N400 to concrete words in the mental image task compared to the surface task and an anterior N700 to concrete words only in the mental image task. In another study, Nittono, Suehiro, and Hori [105] asked participants to rate imageability and found that concrete words elicited both a larger N400 and a later going negativity (N800) than abstract words.

The anterior N400 component has been proposed to reflect processing of visual semantic information in the form of high-level descriptions of the visual properties of concrete objects [107]. The anterior N700 has been proposed to reflect activation in a more frontal brain region, such as the prefrontal cortex, and as such, is implicated in higher cognitive functions such as working memory, i.e., mental images are held in mind to make a judgment [108]. Concreteness effects to words and object working memory have been proposed to have overlapping neural structures. Research supporting this proposal has found suppression of visualization to concrete words by a concurrent (non-semantic) object working memory task, with the requirement of maintaining an object in working memory affecting the amplitude to concrete words [107]. For Experiment 2, we hypothesized that if participants were taking a visualization approach to the concrete word pairs, then even in the absence of a behavioural concreteness effect, a N400 and an anterior N700 concreteness effect would be expected.

## Experiment 2

### Method

#### Participants

The study was approved by the University of Windsor Research Ethics Board (REB #: 16–001). Participants were recruited from February 11, 2016 to March 23, 2016. Twenty-three (six males, 17 females, *M*_*age*_ = 20.4 years, age range: 18–35 years) University of Windsor undergraduate students participated for partial course credit. All participants were at least 18 years of age, had learned English as their first language, were right-handed, and had normal or corrected-to-normal vision. Additionally, all participants were in good health, and none reported neurologic or psychiatric history.

#### Materials

Experiment 2 used the same stimulus set as the iconicity task from Experiment 1.

#### Procedure

**Fig 3:**
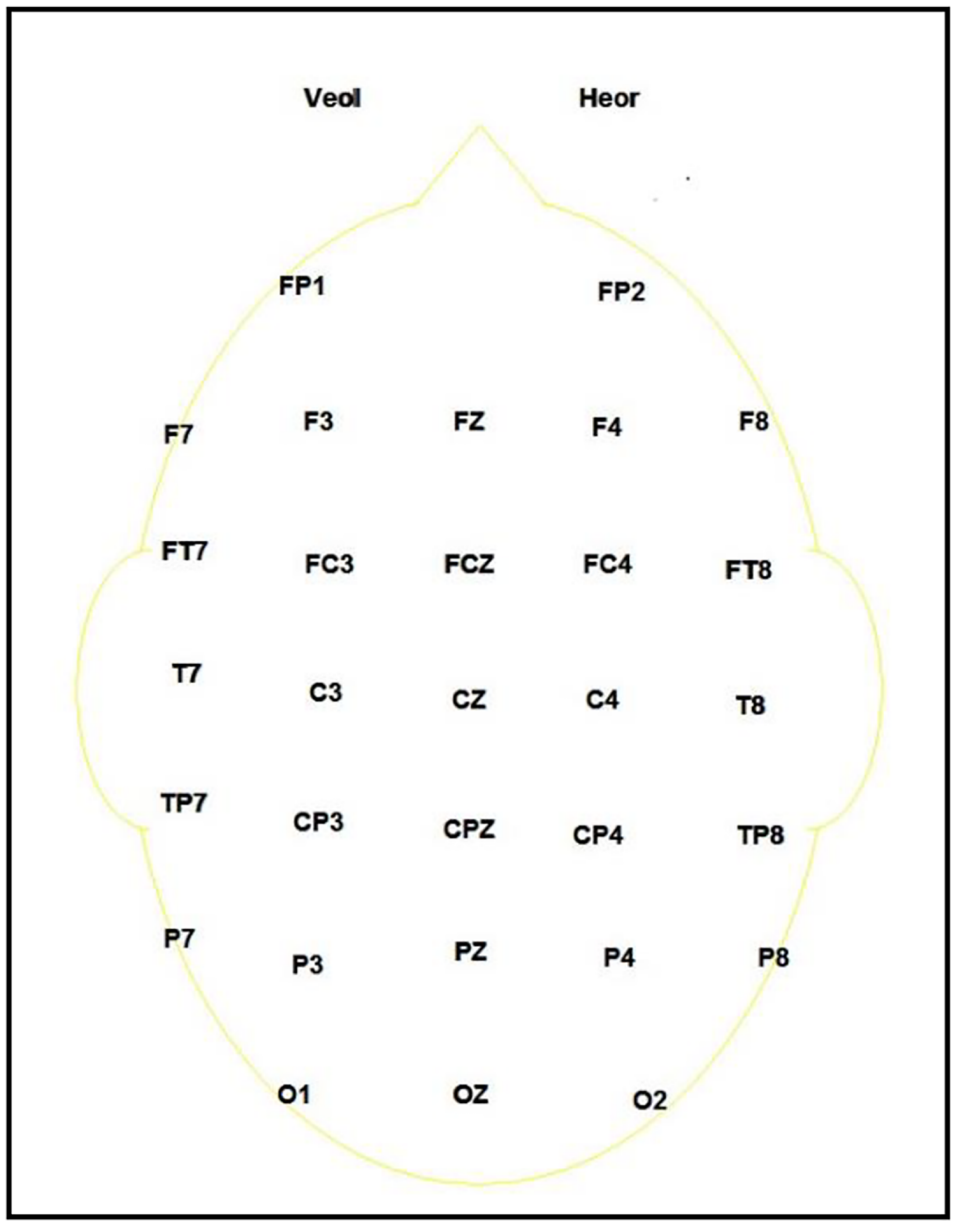
Montage of electrode placements on the scalp.

The procedure was described to participants and written informed consent was obtained. Horizontal eye movements were monitored using an electrode placed 1 cm lateral to the outer canthus of the right eye and vertical eye movements and blinks were monitored by an electrode placed above the center of the left eye. ERP data was recorded using an electrocap from 30 scalp sites (FP1, FP2, F7, F8, F3, F4, FT7, FT8, FC3, FC4, C3, C4, CP3, CP4, TP7, TP8, T7, T8, P3, P4, P7, P8, O1, O2, FZ, FCZ, CZ, CPZ, PZ, OZ) referenced to two electrodes on the left and right mastoids. The ground electrode was located 10 mm anterior to Fz. See Fig 3 for the electrode montage. Scalp and mastoid electrode impedances were maintained below 5 kOhms and eye electrode impedances below 10 kOhms. The data was continuously sampled at a rate of 1000 Hz/channel. The signals were amplified by Neuroscan SynAmps^2^ amplifiers from Compumedics. The data was low-pass filtered (half-amplitude cutoff = 40 Hz, slope = 24 dB/octave). The data was recorded and stored on a computer running Neuroscan Acquire 4.5 software.

Following the set-up, participants were shown a monitor with the ERP signals. Participants were asked to blink and scrunch up their face to show them how signals could be affected by blinks and facial expressions. Participants were then instructed to remain still while completing the task in order to reduce artifacts. Next, a 5-minute baseline was established while participants looked at a blank computer screen with their index fingers positioned on the response keys. The rest of the procedure was identical to the iconicity task from Experiment 1. No participants were assigned to complete the semantic relatedness task.

### Results

#### Behavioural results

Using a minimum accuracy rate of 70%, responses for two concrete word pairs and one participant were removed. The analyses were performed on the remaining data. All incorrect responses, as well as responses faster than 300 ms, were removed, resulting in the removal of 117 observations (6.52% of the remaining data).

As fixed effects, the factors concreteness, iconicity, and semantic neighbours were entered into the model. As random effects, subjects and items were entered into the model. The model was fitted with random slopes for iconicity by item and random slopes for concreteness by subject. After the model was fitted, 36 observations were removed (2.15% of the data). Participant mean logged RTs, SDs, and error rates per condition for the final data set are displayed in Table 5.

**Table 5:** Mean log RTs (with SDs) and average error rates per condition.

Condition	Mean Log RT (ms)	Average Error Rate (%)
**Abstract-Close-Iconic**	7.31 (.51)	3.48
**Abstract-Close-Reverse Iconic**	7.38 (.45)	5.65
**Abstract-Distant-Iconic**	7.34 (.42)	3.91
**Abstract-Distant-Reverse Iconic**	7.46 (.39)	6.52
**Concrete-Close-Iconic**	7.7 (.45)	8.26
**Concrete- Close-Reverse Iconic**	7.76 (.48)	8.7
**Concrete-Distant-Iconic**	7.78 (.44)	5.65
**Concrete-Distant-Reverse Iconic**	7.79 (.43)	10.63

There was a main effect of concreteness, with participants responding faster to abstract stimuli compared to concrete stimuli [*t*(66) = -6.1, *p <* .001]. There was no main effect of either iconicity [*t*(63) = -1.55, *p* = .13] or semantic neighbours [*t*(58) = -1.33, *p* = .19].

For accuracy, the model was fitted with random slopes for iconicity by item. The optimizer bobyqa was used to fit the model. There was a main effect of concreteness [*z* = -5.9, *p <* .001], with concrete stimuli increasing the likelihood of making an error by .14 times ± 1.18 (standard errors). There was a main effect of iconicity [*z* = -2.51, *p* = .012], with reverse-iconic stimuli increasing the likelihood of making an error by .38 times ± 1.04 (standard errors). There was a main effect of semantic neighbours [*z* = 7.04, *p <* .001], with distant neighbours increasing the likelihood of making an error by .13 times ± 1.13 (standard errors).

#### ERP results

**Fig 4:**
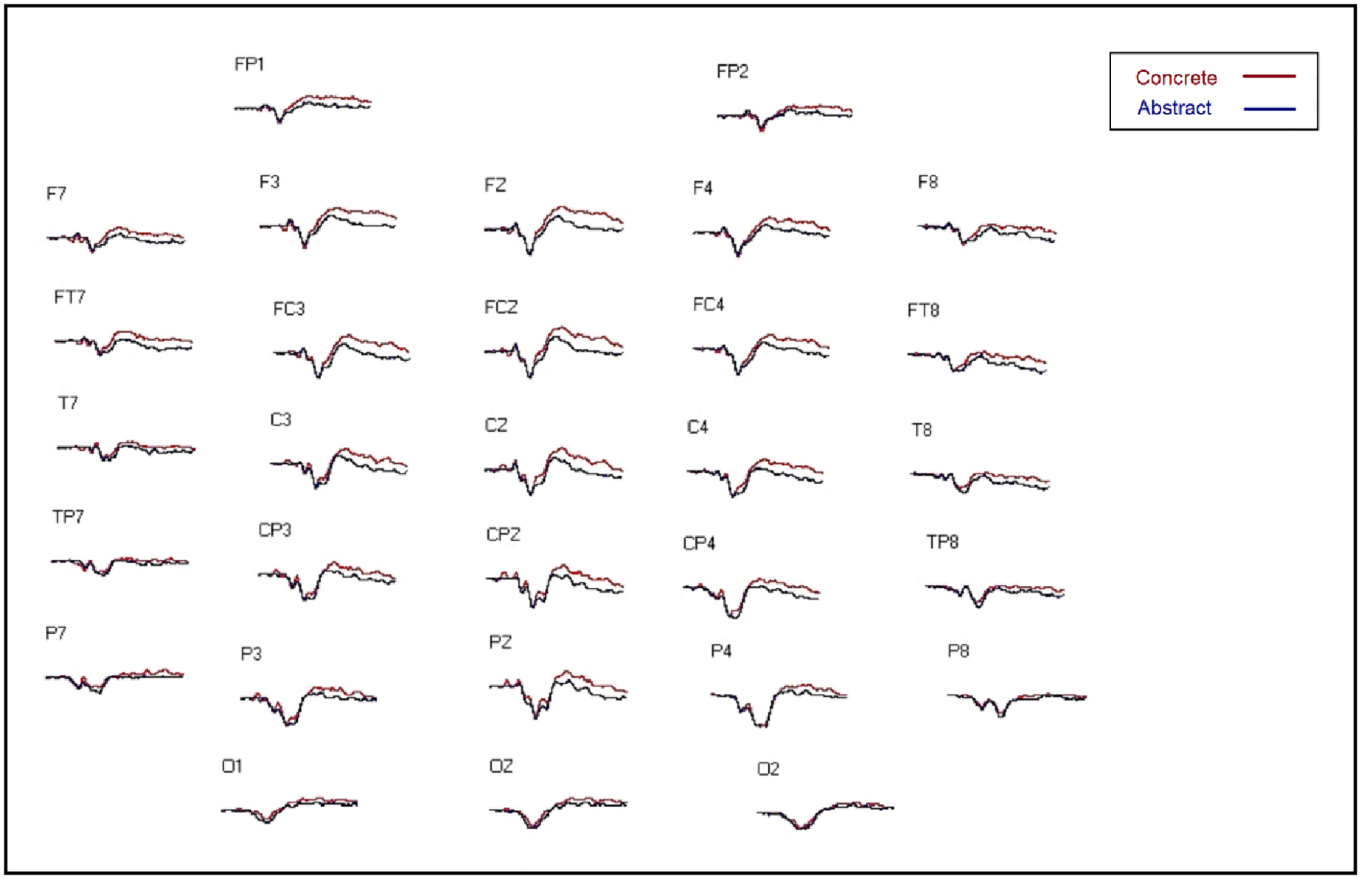
Grand average waveforms for concrete and abstract conditions. Negative amplitudes peak upwards and positive amplitudes peak downwards.

Data was baseline corrected and trials contaminated by eye movements, muscular activity, or electrical noise were excluded from the analyses. Grand average waveforms for concrete and abstract conditions across all scalp electrodes are presented in Fig 4. For each averaged ERP waveform, amplitude and latency of the N400 (300–500 ms) and N700 (500–800 ms) components were measured using a computer program, ERPScore, which enabled both the automatic scoring of peak amplitude and latency within a predefined time window as well as visual inspection of the average waveform [113]. For every subject, statistical analyses were conducted on the peak amplitude of 14 central and posterior electrode sites (central electrodes: C3, C4, CP3 CP4, T7, T8; posterior electrodes: O1, O2, P3, P4, P7, P8, TP7, TP8) within the N400 epoch and on the peak amplitude of 10 anterior electrode sites (FP1, FP2, F3, F4, F7, F8, FC3, FC4, FT7, FT8) within the N700 epoch. Correct responses were analyzed using repeated measures ANOVAs. Greenhouse-Geisser-corrected p-values are reported due to violations of sphericity common in ERP data [114]. For the N400 epoch, there was a significant interaction between concreteness and electrode site [*F*(1, 22) = 4.41, *p* = .047, partial η^2^ = .17]. Follow-up analyses revealed that concrete stimuli were associated with a more negative waveform than were abstract stimuli toward more central scalp locations [*t*(22) = 2.75, *p* = .012]. The voltage difference between concrete and abstract stimuli was not significant at posterior scalp locations[*t*(22) = 1.99, *p* = .059]. There were no significant main effects of iconicity [*F*(1, 22) = .025, *p* = .88] or semantic neighbours [*F*(1, 22) = .97, *p* = .34] and no significant interactions between these factors and electrode site. For the N700, an omnibus ANOVA of the peak amplitudes showed that overall concrete stimuli were associated with a more negative waveform than were abstract stimuli [*F*(1, 22) = 9.09, *p* = .006, partial η^2^ = .29]. There were no significant main effects of iconicity [*F*(1, 22) = .1, *p* = .76] or semantic neighbours [*F*(1, 22) = .35, *p* = .56]. There were no significant findings with respect to latencies.

### Discussion

The main goal of Experiment 2 was to investigate the neural underpinnings of the reverse concreteness, or abstractness, effect observed in Experiment 1. The iconicity task from Experiment 1 was replicated in an ERP paradigm as the abstractness effect was greater in this task than in the semantic relatedness task. A related goal of Experiment 2 was to test the hypothesis that participants were visualizing the concrete word pairs but not the abstract word pairs. As the N400 is generated in response to concrete words and the N700 is considered to be an index of imagery, we predicted that both components would be greater for the concrete stimuli. Our predictions were supported—both the N400 and the N700 were greater for the concrete word pairs compared to the abstract word pairs. This supports the proposal that in the iconicity task, participants were visualizing the concrete word pairs. This also supports the successful development of our stimulus set in that it measures abstractness while circumventing the confound of concretizing via indirect visualization of abstract words. As RTs were shorter for the abstract word pairs, there was a dissociation between RTs and ERP waveforms (see [90] for another example), with the outcome of behavioural abstractness with neural concreteness. This demonstrates that the same neural activity can manifest differently based on task demands.

## General discussion

The present investigation was a two-part study with Experiment 1 testing the *symbol interdependency hypothesis* with both concrete and abstract stimuli (and a novel symbolic factor, i.e., semantic neighbours from WINDSORS) and Experiment 2 following up on an abstractness finding using ERPs. The stimulus set developed for the present study activated the relationship between the word pairs as opposed to activation at the level of the individual words—this removed the confound of concretizing abstract words. The results of Experiment 1 supported the *symbol interdependency hypothesis*. The symbolic factor (i.e., semantic neighbourhood distance) was recruited for the task tapping symbolic relations (i.e., semantic relatedness task) and the embodied factor (i.e., iconicity) was recruited for the task tapping embodied relations (i.e., iconicity task). Across tasks, and especially for the iconicity task, abstract word pairs had shorter RTs compared to concrete word pairs. We proposed a time-costly (two-steps) visualization approach in the iconicity task for concrete word pairs and a time-efficient (one step) emotional valence approach for abstract word pairs. Experiment 2 replicated the iconicity task in an ERP study and found greater N400 and N700 (an index of imagery) amplitudes for the concrete word pairs compared to the abstract word pairs, supporting the proposal that participants were taking a visualization approach to the concrete word pairs.

In the introduction of this paper, we summarized various word processing theories. The results of Experiment 1 map onto the *symbol interdependency hypothesis* and the results of both Experiment 1 and Experiment 2 map onto Vigliocco et al.’s [98] *theory of embodied abstract semantics*, in which sensory-motor information contributes to understanding of concrete words and emotional information contributes to understanding of abstract words. This theory is consistent with our proposal that for the iconicity task, participants took a visualization approach to the concrete stimuli and an emotional valence approach to the abstract stimuli. While Paivio’s [56] *dual coding theory* has typically been cited to explain the concreteness effect, Paivio [115] recently described that the *dual coding theory* can allow for abstractness effects depending on the stimuli and task. Similarly, we are not reporting abstractness effects as an opposition to concreteness effects. Rather, we are proposing that the type of effect that is observed depends on the stimuli and the nature of the task. The same stimuli characteristics, e.g., visualization, that facilitate RTs in one task may hinder them in another. We also propose that the same neural activity, e.g., N700, may be facilitating or hindering for RTs depending on the nature of the task. Danguecan and Buchanan [116] echoed this argument, finding task-specific effects in the semantic processing of concrete and abstract words.

Some limitations to the present study include the scope of factors and tasks used. Future research can extend the findings of this study even further to other types of symbolic factors, such as different lexical co-occurrence models. Moreover, future research should incorporate a gradient of tasks, with tasks used in the present study serving as a starting point. Such an examination could provide useful constraints on the *symbol interdependency hypothesis*. Future research can also examine the role of individual sensorimotor experience and individual cultural experience in producing the iconicity effect for concrete and abstract stimuli, respectively. For example, some individuals may have a greater sensorimotor experience with birds being on the ground as opposed to in the air. With respect to abstract relationships, some cultures may view guests as being more powerful than their hosts. According to the embodied cognition theory, we would expect individual experience to play a role. In contrast, symbolic theories would argue that linguistic experience would override the effect of individual sensorimotor or cultural experiences. Finally, while our stimulus set did not control for age of acquisition and it could be considered as a limitation and an extraneous variable that is producing the effect in this study, the RTs and errors were in the reverse direction to support such a claim: Abstract words, while acquired later, had shorter RTs and fewer errors.

## Supporting information

S1 Appendix Target word pairs (with semantic neighbourhood distance) with their lengths (Len.) frequencies (Freq.), and age of acquisition (AoA). (PDF)

S2 Appendix Filler word pairs for the semantic relatedness task in Experiment 1. (PDF)

S3 Appendix Task instructions. (PDF)

S1 Dataset Experiment 1 RT data. (CSV)

S2 Dataset Experiment 1 accuracy data. (CSV)

S3 Dataset Experiment 2 RT data. (CSV)

S4 Dataset Experiment 2 accuracy data. (CSV)

S5 Dataset Experiment 2 ERP N400 data. (SAV)

S6 Dataset Experiment 2 ERP N700 data. (SAV)
