# Supplementary material for: A test of the symbol interdependency hypothesis with both concrete and abstract stimuli
Source: PLoS One. 2018 Mar 28;13(3):e0192719. doi: 10.1371/journal.pone.0192719 (PMC5873929; doi:10.1371/journal.pone.0192719)
Supplement: S1 Appendix — (PDF) [file pone.0192719.s001.pdf]

S1 Appendix. Target Word Pairs (with Semantic Neighbourhood Distance) with their Lengths (Len.) Frequencies (Freq.), and Age of Acquisition (AoA).

Condition	Word Pair	Len.	Freq.	AoA
**Concrete-Close**	NOSE(9) – TONGUE(22)	10	61.79	4.47
	FLAME(10) – CANDLE(24)	11	26.98	6.25
	HIKER(7) – TRAIL(20)	10	32.06	8.50
	KNEE(2) – ANKLE(2)	9	18.30	4.89
	BRIDGE(25) – LAKE(26)	10	61.34	5.58
	CASTLE(42) – MOAT(14)	10	30.07	9.65
	STOVE(3) – OVEN(3)	9	12.81	5.67
	SHOWER(5) – TUB(17)	9	11.92	4.72
	LID(4) – TRAY(3)	7	10.99	6.05
	LUNGS(32) – STOMACH(27)	12	19.27	7.16
	MOUSTACHE(2) – BEARD(7)	14	17.69	5.40
	JOCKEY(38) – HORSE(49)	11	81.25	8.28
	JACKET(19) – TROUSERS(2)	14	21.62	7.89
	SHIRT(9) – PANTS(4)	10	22.00	3.53
	ROOF(20) – FLOOR(48)	9	94.35	5.00
	CHIMNEY(11) – FIREPLACE(3)	16	12.74	7.37
	MOUTH(25) – THROAT(11)	11	99.39	5.09
	TRAIN(22) – RAILROAD(49)	13	55.24	6.06
	JEANS(4) – SHOES(6)	10	23.29	5.26
	SHOULDERS(8) – HIPS(6)	13	43.09	6.17
**Concrete-Distant**	HORN(679) – TAIL(506)	8	33.52	4.84
	FOAM(3149) – BEER(3107)	8	19.98	6.15
	HOOD(1730) – ENGINE(2598)	10	25.65	6.28
	DESK(422) – CARPET(361)	10	34.11	6.05
	BOOT(797) – HEEL(866)	8	13.26	7.85
	SEAT(1881) – PEDALS(1879)	10	42.42	6.50
	BRANCH(945) – ROOT(625)	10	38.18	5.94
	AIRPLANE(2214) – CAR(2162)	11	81.03	3.94
	PAPER (3633) – CLIPBOARD(2801)	14	86.41	7.76
	HAT(904) – BELT(985)	7	54.14	4.62
	FLOWER(209) – VASE(374)	10	26.58	7.89
	HANDLE(933) – BUCKET(601)	12	26.23	6.30
	MODEL(2460) – RUNWAY(3040)	11	39.28	8.35
	SHEET(506) – MATTRESS(363)	13	19.90	5.33
	FERRY(935) – OCEAN(932)	10	23.42	8.00
	FROTH(2078) – COFFEE(3271)	11	28.45	12.56
	CART(272) – WHEELS(284)	10	21.55	6.16
	BALCONY(1388) – LAWN(1399)	11	13.55	8.10
	SKY(2112) – GRASS(2750)	8	83.85	4.17
	FLAG(665) – POLE(479)	8	24.97	5.63

**Abstract-Close**	COACH(14) – PLAYER(22)	11	42.13	6.89
	JOY(29) – SORROW(8)	9	60.59	8.42
	ABUNDANT(8) – SCARCE(7)	14	16.41	12.84
	TEACHER(11) – STUDENT(6)	14	51.82	5.94
	ANGEL(15) – DEVIL(17)	10	40.73	5.00
	POSITIVE(2) – NEGATIVE(2)	16	41.44	8.11
	ACCEPT(8) – REJECT(4)	12	47.41	9.53
	LANDLORD(4) – TENANT(3)	14	16.42	10.33
	LEND(4) – BORROW(2)	10	17.25	8.45
	VICTORY(2) – DEFEAT(3)	13	35.89	8.74
	BRIGHT(26) – DIM(44)	9	65.40	7.06
	HOST(30) – GUEST(40)	9	34.90	8.05
	CLEAN(19) – DIRTY(46)	10	52.14	4.55
	AGREE(11) – DISAGREE(6)	13	39.86	8.37
	SAFETY(29) – DANGER(29)	12	72.18	5.84
	INCREASE(2) – DECREASE(5)	16	50.54	8.56
	MARRIAGE(3) – DIVORCE(3)	15	53.87	8.90
	FAST(2) – SLOW(2)	8	81.92	4.15
	EXCITEMENT(48) – BOREDOM(13)	17	28.11	7.68
	SMOOTH(2) – ROUGH(3)	11	47.22	6.21
**Abstract-Distant**	PEACE(258) – VIOLENCE(225)	13	81.90	6.39
	OWNER(1306) – PET(1035)	8	28.29	7.50
	SUCCEED(898) – FAIL(998)	11	33.98	8.16
	HEALTHY(1546) – SICK(1338)	11	51.09	7.61
	BOSS(938) – EMPLOYEE(736)	12	20.90	7.84
	ACHIEVEMENT(2088) – FAILURE(2343)	18	38.25	8.80
	CONFIDENT(525) – ARROGANT(295)	17	14.59	9.95
	FIX(324) – BREAK(555)	8	64.84	5.30
	ALLY(1373) – ENEMY(1519)	9	35.16	9.61
	GUARD(2095) – PRISONER(2495)	13	43.18	8.00
	THERAPIST(574) – CLIENT(1005)	15	17.80	12.05
	INTELLIGENT(1892) – STUPID(1167)	17	31.07	8.28
	GAIN(305) – LOSS(394)	8	68.13	7.11
	BLESS(522) – CURSE(992)	10	20.20	7.47
	BOLD(2797) – MEEK(1665)	8	17.40	9.70
	STRAIGHT(800) – CROOKED(1353)	15	64.70	6.80
	FRESH(2402) – STALE(1070)	10	51.82	7.61
	PURE(685) – TAINTED(478)	11	31.72	9.84
	MANAGER(498) – CASHIER(673)	14	51.86	9.40
	BEAUTY(1477) – UGLY(1094)	10	67.75	5.05
