# Supplementary material for: A test of the symbol interdependency hypothesis with both concrete and abstract stimuli
Source: PLoS One. 2018 Mar 28;13(3):e0192719. doi: 10.1371/journal.pone.0192719 (PMC5873929; doi:10.1371/journal.pone.0192719)
Supplement: S2 Appendix — (PDF) [file pone.0192719.s002.pdf]

S2 Appendix. Filler Word Pairs for the Semantic Relatedness Task in Experiment 1.

VACUUM – PURPLE
WOMAN – CURTAIN
FIRE – MONKEY
BLOOD – GRASS
MONEY – UMBRELLA
COMPUTER – SHOES
BURGER – LAMP
SILLY – BLUE
RELAX – MIRROR
CUTLERY – FORGET
BOOK – SNOW
PAIN – YELLOW
RUDE – LOTION
HEAT – PENCIL
PILLOW – TALL
ALPHABET – UPSET
ANIMAL – FOLDER
GAME – LIPSTICK
GARBAGE – BIRTHDAY
WAGON – SOFT
SQUARE – PRAY
FIGHT – OCEAN
MUSCLE – CLOCK
TEETH – STOP
PARENT – WIND
POLICE – HAIR
MOVIE – HELP
CELEBRITY – SLEEP
BRAIN – GHOST
SECRET – APPLE
PAINT – BREAKFAST
DELICIOUS – WIRE
JAIL – TREE
PASTA – BEAR
HONEY – MATH
MAGNET – SOUR
NATURE – FAMOUS
KITCHEN – TRIANGLE
LOCK – FUNNY
MEMORY – NOISY
YARD – AWKWARD
FUNERAL – SOCCER
CIRCUS – FORGIVE
DIARY – KIDNEY
TITLE – WATER
LEAF – CRY
ADVENTURE – SELFISH
WITTY – TENT
TIRED – GOLD
CALCULATOR – ICE
SMOKE – TIE
ROBOT – LEATHER
CHAMPION – ITCHY
CHILD – BRICK
BANANA – JEALOUS
SPOILED – CLIMB
TURBAN – BENCH
PIANO – CANE
HOPEFUL – CHEAP
RAIN – HUNGRY
WOOD – DOG
FRAGRANCE – LONG
LIAR– CEREAL
KING – SOAP
TABLE – DESPAIR
SONG – POISON
PILOT – BABY
LIBRARY – SWEET
BIRD – COUCH
HOSTILE – PROFIT
TEMPER – FRUIT
TIME – SHAME
TREASURE – SHY
DENTIST – ISLAND
WOLF – FANCY
MOTOR – COOKIE
LIGHTS – FRAIL
STRING – JOLLY
BOX – HANDSOME
FUEL – OFFICE
