# Supplementary material for: A test of the symbol interdependency hypothesis with both concrete and abstract stimuli
Source: PLoS One. 2018 Mar 28;13(3):e0192719. doi: 10.1371/journal.pone.0192719 (PMC5873929; doi:10.1371/journal.pone.0192719)
Supplement: S3 Appendix — (PDF) [file pone.0192719.s003.pdf]

S3 Appendix. Task Instructions.

Instructions for the Semantic Relatedness Task

Please indicate as soon as possible whether the pair of words are related in meaning or not by pressing “yes” = related and “no” = unrelated. Sometimes you will see opposites such as *plus* and *minus* and these are considered to be related. When word pairs are unrelated, they will not bear any obvious relationship to one another. You should not have to think of ways to relate the words. Your judgments should be intuitive. Since this is a reaction time experiment, we want you to work as fast as you can – but not at the expense of accuracy. You should use both index fingers to make your responses.

Instructions for the Iconicity Task

Please indicate as soon as possible whether the iconicity of the pair of words is correct or incorrect by pressing “yes” = correct and “no” = incorrect.

Example #1:	Example #2:
POT	DOCTOR
PLANT	PATIENT
The answer is incorrect.	The answer is correct.

Iconicity refers to whether the positions of the words match how they appear in real life. For example, when you think of a pot and a plant, you would expect to see the pot on the bottom, and the plant on top. Because this example has the word *pot* on top and *plant* on the bottom, it is incorrect. In the second example, we are not talking about physical objects anymore, but about power. Doctors are typically considered to have more power than their patients. Because this example shows the word *doctor* on top and *patient* on the bottom, it is correct. We are not asking you to make moral judgments, instead, consider how these concepts stereotypically appear. We also expect happy concepts to be at the top and sad concepts to be at the bottom, so keep these relationships in mind when making your judgments. Since this is a reaction time experiment, we want you to work as fast as you can – but not at the expense of accuracy. You should use both index fingers to make your responses.
